# Association between environmental factors and current asthma, rhinoconjunctivitis and eczema symptoms in school-aged children from Oropeza Province – Bolivia: a cross-sectional study

**DOI:** 10.1186/1476-069X-12-95

**Published:** 2013-11-05

**Authors:** María Teresa Solis-Soto, Armando Patiño, Dennis Nowak, Katja Radon

**Affiliations:** 1 Center for International Health, Ludwig-Maximilians-University, Munich, Germany; 2 Departmental Service of Health (SEDES) - Chuquisaca, Bolivia, South America; 3 Institute for Occupational, Social and Environmental Medicine, Occupational and Environmental Epidemiology & Net Teaching Unit, University Hospital Munich (LMU), Munich, Germany

**Keywords:** Asthma, Rhinoconjunctivitis, Eczema, Children, Environmental factors

## Abstract

**Background:**

In recent years, the prevalence of asthma, rhinoconjunctivitis and eczema symptoms in childhood has considerably increased in developing countries including Bolivia, possibly due to changes in lifestyle, environmental and domestic factors. This study aimed to assess the association between environmental factors and asthma, rhinoconjuctivitis and eczema symptoms in school-aged children from Oropeza Province in Chuquisaca, Bolivia.

**Methods:**

A cross-sectional study was performed in 2340 children attending the fifth grade in 36 randomly selected elementary schools in Oropeza province. The prevalence of symptoms was determined using the International Study of Asthma and Allergies in Childhood (ISAAC) questionnaire. Environmental factors were assessed by the ISAAC environmental questionnaire including questions related to exposure to pets, farm animals, indoor and outdoor pollution, presence of disease vectors at home and precarious household conditions. Generalized linear mixed-effects models were adjusted for age, sex and place of living.

**Results:**

Thirty seven percent of children reported that at least one of their parents smoked at home. Wood or coal was used as cooking fuel in 19% of the homes and 29% reported intense truck traffic on the street where they lived. With respect to hygiene conditions, 86% reported exposure to dogs, 59% exposure to cats and 36% regular contact to farm animals. More than one precarious household condition was reported by 8% of children. In the adjusted model exposure to dog (adjusted OR 1.4; CI 95% 1.0-1.9), cat (1.2; 1.0-1.5), farm animals (1.5; 1.2-1.8); intense truck traffic (1.3; 1.0-1.6), parents smoking at home (1.2; 1.0-1.5), presence of disease vectors at home (fourth quartile vs. first quartile: 1.6; 1.2-2.3) and two or more precarious household conditions (1.5; 1.0-2.2) were significantly associated with rhinoconjunctivitis symptoms. The associations were similar for asthma and eczema symptoms; however it did not reach the level of statistical significance for all items.

**Conclusion:**

Our results support previous findings reported for poor communities especially in Latin America, showing that lower hygiene conditions did not have protective effect against asthma and rhinoconjunctivitis and eczema symptoms.

## Background

Asthma, rhinoconjunctivitis and eczema in childhood are common chronic diseases world-wide [1,2]. The prevalence of these diseases has considerably increased worldwide in recent years, especially in some regions of Latin America [3-5]. The causes of this increase are not fully understood, some hypotheses are related to changes in lifestyle and environmental and domestic factors [6,7] that interact with the immune system in early stages of life [8].

The International Study of Asthma and Allergies in Childhood (ISAAC), the largest worldwide collaborative respiratory research project [9], reported a wide variability of these diseases in the same region and even in the same countries [4,5,10]. The Latin American region had presented both the lowest and the highest prevalences of asthma and allergies compared with the rest of the world [11]. It seems that this variability is independent of geographic, climate, cultural, ethnic or language aspects [12].

The hygiene hypothesis, proposed for the first time in 1989 by Strachan, suggests that the increase in the prevalence of allergic diseases is linked to a decrease in exposure to germs [13]. This hypothesis appears to be related to the increase in allergic diseases in developed countries [14]. In Latin America it has been shown that the prevalence of asthma and allergies in some low-income-resourced countries was associated with low hygienic standards and was similar or even higher than in industrialized ones [2,15,16]. At the same time, environmental exposures which might trigger asthma (e.g., traffic, cooking fuels) are frequent in many Latin American houses [17]. In these countries, such indoor and outdoor environmental factors vary largely between urban and rural areas and by socioeconomic class [12,18]. Studies addressing the link between these factors and asthma and allergies are so far scarce in Latin America. The knowledge of – especially – the association between modifiable environmental factors and asthma and allergies could help to implement prevention strategies which are urgently needed to reduce the burden of these diseases in Latin America. Bolivia is especially suitable for such types of studies as indoor and outdoor environmental exposures and hygiene conditions vary a lot by socioeconomic class, between urban and rural populations as well as between cultural groups.

Therefore, this study aimed to assess the associations of possible modifiable environmental factors and asthma, rhinoconjuctivitis and eczema symptoms in school-aged children from Oropeza Province in Chuquisaca, Bolivia.

## Methods

### Design

From July to December 2011, a cross-sectional study was conducted using the ISAAC methodology [9] in the Oropeza Province, located in the north western part of Chuquisaca, Bolivia.

### Setting

Oropeza is a province in central Bolivia with three sections (Sucre, Yotala and Poroma), with a population of 336 218 and nearly 20% living in rural areas. Forty six percent of the population is living in poverty, 65% of the population 15 years and older identify themselves with some ethnic group (Aymara, Quechua, Guaraní, among others) and nearly 40% of the population over 6 years old speaks some native language (mainly quechua) [19]. The province has warm and dry climate, temperature ranges between 15 - 20°C and altitude between 2000 and 4000 meters above the sea level [20].

### Study subjects

A list of 185 schools with at least 20 pupils at the fifth grade was provided by the Regional Education Service (schools with less than 20 pupils were excluded for feasibility reasons). Forty-three schools were randomly selected from this list and 36 schools accepted to participate (10 schools in the rural area with less than 2000 inhabitants). In each of the sampled schools, the whole fifth grade student population was studied. Spanish is the first language of instruction in all schools.

The study population consisted of 2584 children attending the fifth grade of elementary school and 91% of them (2340 children) accepted to participate. The sample size was determined following ISAAC project recommendations considering 1% of significance level and 80% of statistical power [9].

In contrast to ISAAC recommendation [9], this grade was selected due to the high drop-out rate in the higher grades, especially in Bolivian public schools [21], and the difficulty in implementing the ISAAC parental questionnaire due the low adult literacy level in this region [22]. In this region, nearly 90% attended their fifth grade of schooling and the dropout rate is less than 1.5% [23]. Children in this grade are able to understand and respond appropriately; therefore all the study instruments were completed by the children themselves.

### Study instruments and variable definition

The Spanish version of the ISAAC standardized questionnaire assesses 12 months prevalence of asthma, rhinitis and eczema symptoms [24]. For the current analyses the following definitions were used in accordance with the ISAAC methodology:

Asthma symptoms was defined by a positive response to the question: “Have you had wheezing or whistling in the chest in the past 12 months?”.

Rhinoconjunctivitis symptoms was defined by a positive response to both of the following questions:

a) “In the past 12 months, have you had a problem with sneezing, or a runny, or blocked nose when you did not have a cold or the flu?”

b) “In the past 12 months, has this nose problem been accompanied by itchy, watery eyes?”

Current Eczema Symptoms: were defined by a positive response to all of the following three questions:

a) Did you ever have an itchy rash that was coming and going for at least 6 months?

b) Did you have this itchy rash at any time in the last 12 months?

c) Has this itchy rash at any time affected any of the following places: the folds of the elbows, behind the knees, in front of the ankles, under the buttocks, or around the neck, ears, or eyes?

The ISAAC video questionnaire AVQ3.0 (VQ) designed to assess asthma symptoms [25] was implemented according the ISAAC standard recommendations. For the purpose of this study, current asthma symptoms were defined as a positive response to the scene showing “moderate wheeze at rest”. Both the video scene selected and the question from the written questionnaire used for this analysis, have been shown to have the highest degree of agreement evaluating current wheezing [25-27].

Environmental risk factors were assessed with an adapted version of the ISAAC environmental questionnaire (EQ) for 13–14 years old [9]. For the current analysis we considered indoor and outdoor pollution.

Current exposure to dogs, cats or farm animals (cows, goats, sheep, pigs, hens, chickens or turkeys) were defined if children had a dog or a cat at home, or regular contact (at least once a week) with farm animals in the last 12 months.

Environmental tobacco smoke exposure was defined as present if participants reported that their mother or father smoked at home.

Outdoor air pollution was assessed by the frequency of truck traffic on the street where children lived (never, seldom, frequently through the day, and almost the whole day). Intense truck traffic was considered present when truck traffic was reported almost the whole day and this was consistent with other studies [28].

The use of wood or coal as cooking fuel was considered a risk factor for the outcomes under study.

Household conditions precarity was assessed by classifying household by the number of precarious household characteristics (none, one and two or more) according to the Bolivian National Statistical Institute precarious household characteristic definition [29] (precarious floor: soil; precarious walls: cane, palm or trunk; precarious source of water for cooking: river, lake, pump or water tank car; precarious sewage system: surface or septic tank).

Presence of disease vectors at home (fleas, ticks, kissing bugs, mice, bedbugs and flies) was assessed as a proxy of hygiene conditions. For the analysis, the number of types of disease vectors was categorized into quartiles (25^th^ percentile = 1 vector; 50^th^ percentile 2 vectors; 75^th^ percentile ≥ 3 vector).

Finally, the number of item ownership was explored according to the Bolivian National Statistical Institute classification (radio, TV, bicycle, motorbike, car, refrigerator and telephone). For the analysis, the number of item ownership was categorized into quartiles (25^th^ percentile ≤ 3 items; 50^th^ percentile 4 items; 75^th^ percentile 5–6 items).

A pilot study in rural and urban area was performed to assess the feasibility of the study, adequacy of research instruments and facilitate the understanding and implementation in all the study procedure.

### Ethical considerations

The study was approved by the National Research Ethics Committee from San Andres University at La Paz – Bolivia. Permission to administer the questionnaire was obtained from the Regional Education Service (SEDUCA- Chuquisaca). A written informed consent form as well as a letter explaining the importance of the study was sent to parents or legal guardians of the children one week before the visit to the school. Voluntary participation of children was respected.

### Statistical analysis

The data was double-entered using EpiInfo Version. 3.5.3 for Windows. The two sets of records were compared and discrepancies were corrected. Afterwards, Data was exported to R v. 3.0.1 [30] software for statistical analysis.

Intraclass correlation coefficient (ICC) was computed [31] to analyze cluster design effect for each of the outcomes. All the analyses were adjusted by cluster sampling.

Absolute and relative frequencies were calculated to describe the study population.

Crude and adjusted odds ratios (OR) and 95% confidence intervals (CI) were calculated using generalized linear mixed-effects models [32] to estimate the likelihood of having asthma, rhinoconjuctivitis and eczema symptoms, given the presence of potential risk factors. The adjusted models included variables which were statistically significant (P value ≤ 0.05) in any of the bivariate analysis. In addition, age (three categories: ≤ 10, 11 and ≥12 years), place of living (two categories: urban and rural), and sex were included in all analyses a priori.

## Results

The median age of children was 11 years (range 9 to 15 years), 52% were female and 26% lived in rural areas. Thirty seven percent of children reported that at least one of their parents smoked at home. Wood or coal was used as cooking fuel in 19% of the homes.

About one third of the study population reported intense truck traffic on the street where they lived (29%). With regard to hygiene conditions, exposure to dogs (86%) and cats (59%) was common. Regular contact to farm animals was reported by more than one third of the study population (36%). More than one precarious household condition was reported by 29% of children (Table 1).

**Table 1:** Sample characteristics (N = 2340)

**Variables**		**Total**	**Urban**	**Rural**	P value*
		**% (n)**	**% (n)**	**% (n)**	
**Sex**	Female	52.1 (1217)	53.2 (917)	48.9 (300)	0.14
**Age**	9 years	2.8 (64)	3.2 (55)	1.5 (9)	< 0.05
	10 years	40.1 (933)	44.8 (767)	27.1 (166)	
	11 years	41.9 (975)	39.3 (672)	49.4 (303)	
	12 years	11.2 (260)	9.6 (165)	15.5 (95)	
	13 years	3.2 (74)	2.2 (38)	5.9 (36)	
	14 years	0.6 (14)	0.6 (11)	0.5 (3)	
	15 years	0.2 (5)	0.2 (4)	0.2 (1)	
**Environmental factors**					
**Current exposure to dog**		86.2 (2003)	86.4 (1478)	85.8 (525)	0.75
**Current exposure to cat**		59.2 (1375)	58.1 (994)	62.4 (381)	0.09
**Current exposure to farm animals**		36.1 (837)	31.6 (539)	48.9 (298)	< 0.05
**Intense truck traffic**^**1**^		28.6 (655)	29.4 (494)	26.5 (161)	0.32
**Environmental tobacco smoke exposure**		37.1 (868)	31.7 (547)	52.4 (321)	< 0.05
**Cooking fuel**	Wood or coal	18.5 (431)	9.7 (167)	43.1 (264)	< 0.05
**Disease vectors presence at home **^**2 **^**(Median 2; Range 0 to 6)**	None	8.4 (197)	9.0 (155)	6.9 (42)	< 0.05
	1 Vector	39.3 (920)	41.8 (722)	32.3 (198)	
	2 Vectors	23.9 (559)	24.6 (424)	22.0 (135)	
	3 Vectors	16.5 (385)	16.3 (282)	16.8 (103)	
	≥ 4 Vectors	12.0 (279)	8.3 (144)	22.0 (135)	
**Precarious household conditions **^**3**^	None	70.6 (1615)	77.8 (1310)	50.5 (305)	< 0.05
	1	21.2 (484)	17.0 (286)	32.8 (198)	
	≥ 2	8.2 (188)	5.2 (87)	16.7 (101)	
**Number of item ownership**^**4 **^**(Median 4; Range 0 to 7)**	None	0.6 (13)	0.6 (11)	0.3 (2)	< 0.05
	1 item	6.4 (149)	4.7 (82)	10.9 (67)	
	2 items	7.4 (173)	5.5 (95)	12.7 (78)	
	3 items	16.4 (383)	15.3 (264)	19.4 (119)	
	4 items	22.3 (522)	22.1 (382)	22.8 (140)	
	5 items	22.3 (521)	23.6 (408)	18.4 (113)	
	≥ 6 items	24.8 (579)	28.1 (485)	15.3 (94)	
**Symptoms**					
**Current asthma symptoms WQ**^**5**^		17.8 (414)	16.4 (281)	21.7 (133)	0.11
**Current asthma symptoms VQ**^**6**^		6.4 (148)	7.3 (124)	3.9 (24)	0.23
**Current rhinoconjunctivitis symptoms**^**7**^		22.2 (516)	21.5 (369)	24.0 (147)	0.54
**Current eczema symptoms**^**8**^		9.2 (213)	9.5 (161)	8.5 (52)	0.59

*Chi square Test.

^1^Intense truck traffic: Almost the whole day.

^2^Disease vectors: fleas, ticks, kissing bugs, mice, bedbugs, flies.

^3^Precarious floor: soil; Precarious walls: cane, palm or trunk; Precarious source of water for cooking: river, lake, pump or water tank car; Precarious sewage system: to the surface or to a septic tank.

^4^Item ownership: Radio, TV, bicycle, motorbike, car, refrigerator, telephone.

^5^Positive answer to the question: “Have you had wheezing or whistling in the chest during the last 12 months?”in the written questionnaire.

^6^Positive answer to the first scene of the video questionnaire: Moderate wheezing at rest.

^7^Presence of sneezing or a runny or blocked nose, accompanied by itchy watery eyes without a cold or the flu.

^8^Presence of an itchy rash at any time in the past 12 months affecting the following places: the folds of the elbows; behind the knees; in front of the ankles; under the buttocks; or around the neck, ears, or eyes).

Exposure to environmental tobacco smoke, wood or coal as cooking fuel, farm animals and disease vectors and at least one precarious condition was higher among children in rural compared to those in urban areas. Children from rural areas had a higher median age and a lower number of assets as shown in Table 1.

The prevalence of current asthma symptoms based on the written questionnaire was 18% and 6% reported asthma symptoms according to the video questionnaire. Although there were no significant differences between rural and urban areas, we found higher prevalences of asthma symptoms detected by written questionnaire (22%) and rhinoconjunctivitis symptoms (24%) in urban areas in comparison with rural areas (16 and 22% respectively) (Table 1).

The unadjusted and adjusted models showed that current exposure to cats, farm animals, intense truck traffic, parents smoking at home and presence of disease vectors were associated with asthma, rhinoconjunctivitis and eczema symptoms. However, it did not reach the level of statistical significance for all items (Table 2).

**Table 2:** **Association between environmental factors and asthma, rhinoconjunctivitis and eczema symptoms during the past 12 months (unadjusted and adjusted odds ratios with 95**% **confidence intervals) (N = 2340)**

**Variables**		**Current asthma symptoms WQ**^**1**^		**Current asthma symptoms VQ**^**2**^		**Current Rhinoconjunctivitis symptoms**^**3**^		**Current eczema symptoms**^**4**^	
		**Crude OR (95% CI)**	**a OR**^**5 **^**(95% CI)**	**Crude OR (95% CI)**	**a OR**^**5 **^**(95% CI)**	**Crude OR (95% CI)**	**a OR**^**5 **^**(95% CI)**	**Crude OR (95% CI)**	**a OR**^**5 **^**(95% CI)**
**Current dog contact**	Yes^6^	1.36 (0.97–1.92)	1.31 (0.90–1.90)	1.38 (0.79–2.38)	1.23 (0.65–2.31)	1.60 (1.16–2.20)	1.37 (0.97–1.94)	1.08 (0.71–1.65)	0.85 (0.54–1.35)
**Current cat contact**	Yes^6^	1.18 (0.94–1.48)	1.09 (0.86–1.39)	1.48 (1.02–2.15)	1.43 (0.96–2.13)	1.35 (1.09–1.66)	1.22 (0.97–1.52)	1.46 (1.07–1.97)	1.37 (0.99–1.90)
**Current contact to farm animals**	Yes^6^	1.29 (1.03–1.62)	1.19 (0.94–1.51)	1.13 (0.79–1.63)	0.97 (0.66–1.44)	1.71 (1.39–2.10)	1.48 (1.19–1.84)	1.46 (1.09–1.96)	1.31 (0.96–1.79)
**Intense truck traffic7**	Yes^6^	1.24 (0.98–1.57)	1.22 (0.95–1.56)	1.53 (1.06–2.20)	1.53 (1.04–2.25)	1.33 (1.07–1.65)	1.27 (1.01–1.59)	1.44 (1.07–1.95)	1.44 (1.05–1.97)
**Environmental tobacco smoke exposure**	Yes^6^	1.30 (1.04–1.63)	1.23 (0.97–1.56)	1.07 (0.75–1.55)	1.12 (0.76–1.66)	1.31 (1.06–1.61)	1.21 (0.97–1.51)	1.34 (1.00–1.80)	1.30 (0.95–1.77)
**Cooking fuel**	Gas or electricity	1	1	1	1	1	1	1	1
	Wood or coal	1.01 (0.75–1.37)	0.86 (0.62–1.19)	1.80 (1.12–2.89)	1.94 (1.16–3.26)	0.99 (0.75–1.30)	0.81 (0.60–1.11)	0.95 (0.64–1.40)	0.84 (0.54–1.30)
**Presence of disease vectors at home**^**8**^	Quartile 1	1	1	1	1	1	1	1	1
	Quartile 2	1.19 (0.90–1.57)	1.14 (0.86–1.53)	1.02 (0.65–1.60)	1.15 (0.72–1.84)	0.94 (0.72–1.22)	0.88 (0.67–1.16)	0.91 (0.62–1.35)	0.88 (0.59–1.32)
	Quartile 3	1.27 (0.93–1.73)	1.17 (0.85–1.61)	1.09 (0.67–1.79)	1.10 (0.65–1.88)	1.31 (0.99–1.73)	1.25 (0.94–1.68)	1.54 (1.05–2.26)	1.54 (1.03–2.30)
	Quartile 4	1.68 (1.20–2.36)	1.49 (1.04–2.14)	1.81 (1.08–3.03)	2.19 (1.25–3.82)	1.84 (1.35–2.50)	1.64 (1.18–2.30)	1.87 (1.23–2.82)	1.82 (1.15–2.87)
**Precarious household conditions**^**9**^	None	1	1	1	1	1	1	1	1
	1	1.19 (0.91–1.57)	1.22 (0.92–1.62)	1.09 (0.70–1.70)	0.93 (0.57–1.51)	1.03 (0.79–1.33)	1.00 (0.76–1.31)	0.96 (0.67–1.40)	0.92 (0.62–1.36)
	≥ 2	1.31 (0.89–1.92)	1.30 (0.86–1.96)	1.03 (0.53–2.01)	0.94 (0.47–1.89)	1.57 (1.10–2.24)	1.51 (1.03–2.22)	1.24 (0.75–2.04)	1.12 (0.64–1.94)
**Number of item ownership**^**10**^	Quartile 1	1	–	1	–	1	–	1	–
	Quartile 2	0.96 (0.71–1.30)	–	1.12 (0.68–1.84)	–	0.84 (0.63–1.11)	–	1.31 (0.88–1.95)	–
	Quartile 3	0.94 (0.69–1.29)	–	1.23 (0.75–2.02)	–	0.86 (0.65–1.14)	–	1.20 (0.79–1.80)	–
	Quartile 4	1.03 (0.76–1.39)	–	1.06 (0.64–1.75)	–	0.94 (0.71–1.24)	–	1.18 (0.78–1.76)	–

^1^Positive answer to the question: “Have you had wheezing or whistling in the chest during the last 12 months?”in the written questionnaire.

^2^Positive answer to the first scene of the video questionnaire: Moderate wheezing at rest.

^3^Presence of sneezing or a runny or blocked nose, accompanied by itchy watery eyes without a cold or the flu.

^4^Presence of an itchy rash at any time in the past 12 months affecting the following places: the folds of the elbows; behind the knees; in front of the ankles; under the buttocks; or around the neck, ears, or eyes.

^5^Adjusted by sex, age and place of living.

^6^Comparison group: no contact/exposure.

^7^Intense truck traffic: Almost the whole day.

^8^Disease vectors: fleas, ticks, kissing bugs, mice, bedbugs, flies.

^9^Precarious floor: soil; Precarious walls: cane, palm or trunk; Precarious source of water for cooking: river, lake, pump or water tank car; Precarious sewage system: to the surface or to a septic tank.

^10^Item ownership: Radio, TV, bicycle, motorbike, car, refrigerator, telephone.

In other hand only wood or coal as cooking fuel showed a positive association with asthma symptoms (VQ) and two or more precarious household conditions and dog contact showed a positive association with rhinoconjunctivitis symptoms. The results did not vary when stratified by place of residence (Additional file 1: Table S1-4).

## Discussion

In this study, outdoor as well as indoor environmental factors and hygiene conditions at home were associated with an increased risk of asthma and allergies in children living in Oropeza province in Bolivia. This contributes to our understanding of the importance of modifiable environmental factors in the development of asthma and allergies in children in Bolivia.

This is the first study in Bolivia exploring environmental risk factors for asthma and allergy symptoms in a representative sample (response over 91%) of children from urban and rural areas using standardized methods. Another strength of our study is related to the use of symptom-based definition of asthma, rhinoconjunctivitis and eczema in order to avoid major diagnostic differences related to access to medical care, language and medical practice. We preferred to use the 12-months prevalence of symptoms over lifetime prevalence as previous studies have shown that this definition is less prone to recall bias [33].

Although cluster effect was not expected to be large in ISAAC studies [9], methods for correlated data were computed as recommended when cluster sampling is implemented [34]. In our study the cluster effect was relatively small (20, 4, 2 and 0.7% for asthma detected by VQ, asthma detected by WQ, rhinoconjunctivitis and eczema symptoms respectively. We did not find significant modifications in comparison with logistic regression models without considering correlated data.

One limitation of our study is related to the cross-sectional design, with the exposure and outcome being measured simultaneously, thus it is not possible to determine the temporality of the association. Although this design is subject to recall bias, we assume that it was minimized as strategies recommended by ISAAC were considered [9]. The terms asthma, allergy, rhinitis or eczema were not mentioned at any time. Furthermore children, parents or school directors were not informed about study hypotheses in detail to prevent their prompt response on the problems and risk factors being studied.

The ISAAC video questionnaire was described as a sensitive tool for language, culture, or literacy differences to assess asthma symptoms [26,35]. As our study population presents a large range of sociocultural and educational background we implemented both – the video as well as the written questionnaire. We found low agreement in prevalence for asthma symptoms by written compared to video questionnaire (Kappa 0.11; p value <0.05). We found lower prevalence of asthma symptoms when VQ is considered with lower prevalence in rural areas in comparison with urban areas. It could be explained because the video scene chosen to define asthmas symptoms might reflect more severe asthma than the WQ as it demonstrates a child having wheezing symptoms at rest [27,36]. Also it is likely that in our study, the unfamiliarity with the terms used in the WQ could cause overreporting of symptoms especially in the rural population [37] or children could interpret a written question about wheezing differently from its audiovisual presentation [26]. Given the sociocultural and educational background differences in our study population we could assume the results of the video questionnaire are more reliable than written questionnaire.

We found more common exposure to indoor pollution (cooking fuel and tobacco smoke) in rural areas in comparison with urban areas. Even if this situation has been reported for cooking fuel previously [19], there are no much information for tobacco smoking in rural setting in Bolivia. This needs further research to explore urban and rural differences regarding type and quantity cigarettes. The results of this study have pointed out several sources of indoor and outdoor air pollution as risk factors of asthma and allergies in children. It is well known that environmental tobacco smoke is associated with asthma symptoms. A recent systematic review and meta-analysis of 79 prospective studies found that exposure to pre- or postnatal environmental tobacco smoke exposure was associated with a 30% to 70% increased risk of incident wheezing [38]. Although the evidence is not too strong for rhinoconjunctivitis and eczema symptoms [33], our results support this association.

Even though the associations between outdoor air pollution with asthma and allergy symptoms have shown paradoxical results in Latin America [12], our results are consistent with those reported by ISAAC phase 3 showing a positive association between intense truck traffic and asthma, rhinoconjuntivitis and eczema symptoms [39]. It could be explained by the interaction of air pollutants with the immune system, increasing the allergenicity of these pathologies.

Although the prevalence for Asthma (WQ) and rhinoconjunctivitis symptoms reported in our study are within the range reported for 13–14 years old children in Latin America region [5,10], these prevalences were higher in rural areas where poverty is over 80% [19]. Our results support previous findings reported for Latin America [12,40,41], showing that asthma and allergies are associated with poverty and inequality. Poverty is a complex social and economic condition and it is not easy to identify the effects of the many associated environmental and lifestyle factors contributing to the development of asthma and allergies [40]. Poverty is associated with a number of known risk factors for asthma and allergies at individual and neighbourhood level as smoking [33], high indoor and outdoor pollution [42,43], diet [44,45], ethnicity [46], higher GINI index [47], lack of good quality potable water [48], sewer systems [49], and waste collection and disposal [50]. In the same way poverty is linked with poor hygienic conditions. This might explain why our as well as other Latin American studies have shown lack of hygiene [12,40,51], exposure to farm animals [52] and pets [53] as risk factors for asthma and allergies while other studies – mainly European studies [54-56] – have indicated an inverse relationship between low level of hygiene and such diseases. Our findings cast doubts on whether differences in phenotype of asthma, rhinoconjunctivitis and eczema in Latin America [57-59] are really the main reason for the dissimilarity with prevalence in other parts of the world [60]. Furthermore, the high prevalence of asthma and rhinoconjunctivitis found in urban and rural children from a poor locality (with higher prevalence in rural than urban children), dismiss any protection given by low socioeconomic conditions, living in rural setting or exposure to farm animals, against asthma and rhinitis in developing settings. On the contrary, our findings support the concept that in non-affluent regions, poverty and associated conditions appear as important risk factors for a higher prevalence of asthma and allergies. The present study shows that potentially preventable environmental aggressors as tobacco smoke and fuel cooking seems to be main determinants for the higher prevalence of asthma found in rural children.

## Conclusions

Our results suggest that reducing exposure to modifiable risk factors like environmental tobacco smoke, improving housing conditions and hygienic conditions would have a significant positive impact on asthma and allergies morbidity in children in poor communities in Latin America. Intervention studies would be the next step to confirm these findings.

## Abbreviations

ISAAC: International study of asthma and allergies in childhood; VQ: The ISAAC video questionnaire AVQ3.0; EQ: The ISAAC environmental questionnaire; SEDUCA-Chuquisaca: The regional education service; OR: Odds ratio; CI: Confidence intervals.

## Competing interests

No conflicts of interests are reported for this study.

## Authors’ contributions

MTSS participated in the design of the study acquisition of data, performed the statistical analysis and wrote the paper. KR conceived of the study, and participated in its design and coordination and helped to draft the manuscript. All authors revised the manuscript critically for important intellectual content, and approved the final manuscript.

## Supplementary Material

Additional file 1 Additional tables show the predicted models for asthma, rhinoconjunctivitis and eczema symptoms stratified for urban and rural areas. Click here for file
