# Supplementary material for: Association between environmental factors and current asthma, rhinoconjunctivitis and eczema symptoms in school-aged children from Oropeza Province – Bolivia: a cross-sectional study
Source: Environ Health. 2013 Nov 5;12:95. doi: 10.1186/1476-069X-12-95 (PMC4228317; doi:10.1186/1476-069X-12-95)
Supplement: Additional file 1 — Additional tables show the predicted models for asthma, rhinoconjunctivitis and eczema symptoms stratified for urban and rural areas. [file 1476-069X-12-95-S1.doc]

**Additional file 1: Table S1: Association between environmental factors and current asthma symptoms (Written questionnaire) by place of residence (unadjusted and adjusted Odds Ratios with 95%** **Confidence Intervals)** **(N=2340)**

| **Variables** |  | **Current asthma symptoms WQ^1^** | | | |
| --- | --- | --- | --- | --- | --- |
|  |  | **Urban** | | **Rural** | |
|  |  | **Crude OR (95%CI)** | **a OR^2^ (95%CI)** | **Crude OR (95%CI)** | **a OR^2^ (95%CI)** |
| **Current dog contact** | Yes^3^ | 1.27 (0.8-1.9) | 1.17 (0.7-1.9) | 1.62 (0.9-3.0) | 1.58 (0.8-3.1) |
| **Current cat contact** | Yes^3^ | 1.26 (1.0-1.7) | 1.19 (0.9-1.6) | 1.00 (0.7-1.5) | 0.94 (0.6-1.4) |
| **Current contact to farm animals** | Yes^3^ | 1.39 (1.1-1.8) | 1.34 (1.0-1.8) | 1.07 (0.7-1.6) | 0.92 (0.6-1.4) |
| **Intense truck traffic**^4^ | Yes^3^ | 1.21 (0.9-1.6) | 1.19 (0.9-1.6) | 1.35 (0.9-2.1) | 1.33 (0.8-2.1) |
| **Environmental tobacco smoke exposure** | Yes^3^ | 1.27 (1.0-1.7) | 1.22 (0.9-1.6) | 1.31 (0.9-2.0) | 1.30 (0.8-2.0) |
| **Cooking Fuel** | Gas or electricity | 1 | 1 | 1 | 1 |
|  | Wood or coal | 1.02 (0.7-1.6) | 0.92 (0.6-1.5) | 0.90 (0.6-1.4) | 0.82 (0.5-1.3) |
| **Presence of disease vectors at home^5^** | Quartile 1 | 1 | 1 | 1 | 1 |
|  | Quartile 2 | 1.27 (0.9-1.8) | 1.24 (0.9-1.7) | 0.98 (0.6-1.7) | 0.90 (0.5-1.6) |
|  | Quartile 3 | 1.31 (0.9-1.9) | 1.19 (0.8-1.7) | 1.15 (0.6- 2.0) | 1.03 (0.6-1.9) |
|  | Quartile 4 | 1.74 (1.1 (2.7) | 1.62 (1.0-2.6) | 1.45 (0.9-2.5) | 1.32 (0.8-2.3) |
| **Precarious household conditions^6^** | None | 1 | 1 | 1 | 1 |
|  | 1 | 1.23 (0.9-1.7) | 1.30 (0.9-1.9) | 1.09 (0.7-1.7) | 1.14 (0.7-1.8) |
|  | ≥ 2 | 0.95 (0.5-1.7) | 1.05 (0.6-2.0) | 1.58 (0.9-2.7) | 1.55 (0.9-2.8) |
| **Number of item ownership^7^** | Quartile 1 | 1 | - | 1 | - |
|  | Quartile 2 | 1.04 (0.7-1.5) | - | 0.87 (0.5-1.5) | - |
|  | Quartile 3 | 1.08 (0.7-1.6) | - | 0.74 (0.4-1.3) | - |
|  | Quartile 4 | 1.17 (0.8-1.7) | - | 0.83 (0.5-1.5) | - |

^1^ Positive answer to the question: ‘‘Have you had wheezing or whistling in the chest during the last 12 months?’’ in the written questionnaire.

^2^Adjusted by sex and age

^3^ Comparison group: no contact / exposure

^4^Intense truck traffic: Almost the whole day

^5^Disease vectors: fleas, ticks, kissing bugs, mice, bedbugs, flies

^6^ Precarious floor: soil; Precarious walls: cane, palm or trunk; Precarious source of water for cooking: river, lake, pump or water tank car; Precarious sewage system: to the surface or to a septic tank

^7^ Item ownership: Radio, TV, bicycle, motorbike, car, refrigerator, telephone

**Additional file 1: Table S2: Association between environmental factors and current asthma symptoms (Video questionnaire) by place of residence (unadjusted and adjusted Odds Ratios with 95%** **Confidence Intervals)** **(N=2340)**

| **Variables** |  | **Current asthma symptoms VQ^1^** | | | |
| --- | --- | --- | --- | --- | --- |
|  |  | **Urban** | | **Rural** | |
|  |  | **Crude OR (95%CI)** | **a OR^2^ (95%CI)** | **Crude OR (95%CI)** | **a OR^2^ (95%CI)** |
| **Current dog contact** | Yes^3^ | 1.62 (0.8-3.1) | 1.46 (0.7-3.2) | 0.85 (0.3-2.5) | 0.77 (0.2-2.8) |
| **Current cat contact** | Yes^3^ | 1.60 (1.1-2.4) | 1.49 (1.0-2.3) | 1.08 (0.4-2.7) | 1.26 (0.4-3.6) |
| **Current contact to farm animals** | Yes^3^ | 1.36 (0.9-2.0) | 1.20 (0.8-1.8) | 0.52 (0.2-1.3) | 0.44 (0.2-1.2) |
| **Intense truck traffic^4^** | Yes^3^ | 1.55 (1.0-2.3) | 1.55 (1.0-2.3) | 1.54 (0.5-4.4) | 1.59 (0.5-4.9) |
| **Environmental tobacco smoke exposure** | Yes^3^ | 1.10 (0.7-1.6) | 1.09 (0.7-1.7) | 1.09 (0.4-2.9) | 1.20 (0.4-3.5) |
| **Cooking Fuel** | Gas or electricity | 1 | 1 | 1 | 1 |
|  | Wood or coal | 1.98 (1.1-3.4) | 1.74 (1.0-3.2) | 2.06 (0.7-5.9) | 2.20 (0.7-6.6) |
| **Presence of disease vectors at home^5^** | Quartile 1 | 1 | 1 | 1 | 1 |
|  | Quartile 2 | 0.99 (0.6-1.6) | 1.09 (0.7-1.8) | 1.94 (0.4-9.6) | 2.00 (0.4-10.2) |
|  | Quartile 3 | 0.95 (0.6-1.6) | 0.89 (0.5-1.6) | 3.55 (0.8-16.0) | 3.32 (0.7-16.4) |
|  | Quartile 4 | 1.84 (1.0-3.3) | 1.98 (1.0-3.8) | 3.70 (0.9-15.5) | 5.19 (1.1-23.5) |
| **Precarious household conditions^6^** | None | 1 | 1 | 1 | 1 |
|  | 1 | 1.03 (0.6-1.7) | 0.80 (0.4-1.4) | 1.23 (0.5-3.3) | 1.16 (0.4-3.3) |
|  | ≥ 2 | 1.68 (0.8-3.5) | 1.44 (0.7-3.1) | 0.41 (0.1-1.6) | 0.35 (0.1-1.5) |
| **Number of item ownership^7^** | Quartile 1 | 1 | - | 1 | - |
|  | Quartile 2 | 0.95 (0.6-1.6) | - | 3.02 (0.8-11.9) | - |
|  | Quartile 3 | 0.93 (0.5-1.6) | - | 7.44 (1.8-30.5) | - |
|  | Quartile 4 | 0.95 (0.6-1.6) | - | 1.35 (0.1-14.9) | - |

^1^ Positive answer to the first scene of the video questionnaire: Moderate wheezing at rest

^2^Adjusted by sex and age

^3^ Comparison group: no contact / exposure

^4^Intense truck traffic: Almost the whole day

^5^Disease vectors: fleas, ticks, kissing bugs, mice, bedbugs, flies

^6^ Precarious floor: soil; Precarious walls: cane, palm or trunk; Precarious source of water for cooking: river, lake, pump or water tank car; Precarious sewage system: to the surface or to a septic tank

^7^ Item ownership: Radio, TV, bicycle, motorbike, car, refrigerator, telephone

**Additional file 1: Table S3: Association between environmental factors and current rhinoconjunctivitis symptoms by place of residence (unadjusted and adjusted Odds Ratios with 95%** **Confidence Intervals)** **(N=2340)**

| **Variables** |  | **Current Rhinoconjunctivitis symptoms^1^** | | | |
| --- | --- | --- | --- | --- | --- |
|  |  | **Urban** | | **Rural** | |
|  |  | **Crude OR (95%CI)** | **a OR^2^ (95%CI)** | **Crude OR (95%CI)** | **a OR^2^ (95%CI)** |
| **Current dog contact** | Yes^3^ | 1.45 (1.0-2.1) | 1.15 (0.8-1.7) | 2.13 (1.1-4.1) | 2.17 (1.1-4.4) |
| **Current cat contact** | Yes^3^ | 1.40 (1.1-1.8) | 1.31 (1.0-1.7) | 1.21 (0.8-1.8) | 1.01 (0.7-1.6) |
| **Current contact to farm animals** | Yes^3^ | 1.82 (1.4-2.3) | 1.62 (1.3-2.1) | 1.46 (1.0-2.2) | 1.15 (0.8-1.8) |
| **Intense truck traffic**^4^ | Yes^3^ | 1.34 (1.0-1.7) | 1.30 (1.0-1.7) | 1.11 (0.7-1.7) | 1.20 (0.8-1.9) |
| **Environmental tobacco smoke exposure** | Yes^3^ | 1.24 (1.0-1.6) | 1.12 (0.9-1.5) | 1.32 (0.9-2.0) | 1.54 (1.0-2.3) |
| **Cooking Fuel** | Gas or electricity | 1 | 1 | 1 | 1 |
|  | Wood or coal | 1.20 (0.9-1.5) | 1.00 (0.7-1.5) | 0.77 (0.5-1.2) | 0.63 (0.4-1.0) |
| **Presence of disease vectors at home^5^** | Quartile 1 | 1 | 1 | 1 | 1 |
|  | Quartile 2 | 1.03 (0.8-1.4) | 0.96 (0.7-1.3) | 0.66 (0.4-1.2) | 0.62 (0.3-1.1) |
|  | Quartile 3 | 1.38 (1.0-1.9) | 1.28 (0.9-1.8) | 1.09 (0.6-1.9) | 1.06 (0.6-1.9) |
|  | Quartile 4 | 1.62 (1.1-2.4) | 1.43 (0.9-2.2) | 1.89 (1.1-3.1) | 1.90 (1.1-3.3) |
| **Precarious household conditions^6^** | None | 1 | 1 | 1 | 1 |
|  | 1 | 1.14 (0.8-1.6) | 1.11 (0.8-1.5) | 0.84 (0.5-1.3) | 0.86 (0.5-1.4) |
|  | ≥ 2 | 1.59 (1.0-2.6) | 1.61 (0.9-2.7) | 1.44 (0.8-2.5) | 1.40 (0.8-2.5) |
| **Number of item ownership^7^** | Quartile 1 | 1 | - | 1 | - |
|  | Quartile 2 | 0.92 (0.7-1.3) | - | 0.69 (0.4-1.2) | - |
|  | Quartile 3 | 0.93 (0.7-1.3) | - | 0.70 (0.4-1.2) | - |
|  | Quartile 4 | 0.92 (0.7-1.3) | - | 1.17 (0.7-2.1) | - |

^1^ Presence of sneezing or a runny or blocked nose, accompanied by itchy watery eyes without a cold or the flu.

^2^Adjusted by sex and age

^3^ Comparison group: no contact / exposure

^4^Intense truck traffic: Almost the whole day

^5^Disease vectors: fleas, ticks, kissing bugs, mice, bedbugs, flies

^6^ Precarious floor: soil; Precarious walls: cane, palm or trunk; Precarious source of water for cooking: river, lake, pump or water tank car; Precarious sewage system: to the surface or to a septic tank

^7^ Item ownership: Radio, TV, bicycle, motorbike, car, refrigerator, telephone

**Additional file 1: Table S4: Association between environmental factors and current eczema symptoms by place of residence (unadjusted and adjusted Odds Ratios with 95%** **Confidence Intervals)** **(N=2340)**

| **Variables** |  | **Current eczema symptoms^1^** | | | |
| --- | --- | --- | --- | --- | --- |
|  |  | **Urban** | | **Rural** | |
|  |  | **Crude OR (95%CI)** | **a OR^2^ (95%CI)** | **Crude OR (95%CI)** | **a OR^2^ (95%CI)** |
| **Current dog contact** | Yes^3^ | 1.16 (0.7-1.9) | 0.89 (0.5-1.5) | 0.90 (0.4-2.0) | 0.82 (0.3-2.0) |
| **Current cat contact** | Yes^3^ | 1.54 (1.1-2.2) | 1.44 (1.0-2.1) | 1.23 (0.7-2.3) | 1.12 (0.6-2.2) |
| **Current contact to farm animals** | Yes^3^ | 1.51 (1.1-2.1) | 1.30 (0.9-1.9) | 1.40 (0.8-2.5) | 1.27 (0.7-2.4) |
| **Intense truck traffic**^4^ | Yes^3^ | 1.74 (1.3-2.4) | 1.73 (1.2-2.5) | 0.70 (0.3-1.4) | 0.70 (0.3-1.5) |
| **Environmental tobacco smoke exposure** | Yes^3^ | 1.74 (1.2-2.4) | 1.35 (0.9-1.9) | 1.12 (0.6-2.0) | 1.17 (0.6-2.2) |
| **Cooking Fuel** | Gas or electricity | 1 | 1 | 1 | 1 |
|  | Wood or coal | 1.00 (0.6-1.7) | 0.81 (0.4-1.5) | 0.96 (0.5-1.8) | 0.94 (0.5-1.8) |
| **Presence of disease vectors at home^5^** | Quartile 1 | 1 | 1 | 1 | 1 |
|  | Quartile 2 | 1.08 (0.7-1.7) | 1.03 (0.7-1.6) | 0.46 (0.2-1.2) | 0.47 (0.2-1.3) |
|  | Quartile 3 | 1.95 (1.3-3.0) | 1.88 (1.2-2.9) | 0.61 (0.2-1.6) | 0.63 (0.2-1.7) |
|  | Quartile 4 | 1.90 (1.1-3.3) | 1.76 (1.0-3.2) | 1.59 (0.8-3.2) | 1.42 (0.7-3.1) |
| **Precarious household conditions^6^** | None | 1 | 1 | 1 | 1 |
|  | 1 | 1.21 (0.8-1.9) | 1.11 (0.7-1.7) | 0.63 (0.3-1.3) | 0.68 (0.3-1.5) |
|  | ≥ 2 | 0.98 (0.5-2.1) | 0.83 (0.4-1.9) | 1.48 (0.7-3.1) | 1.32 (0.6-3.0) |
| **Number of item ownership^7^** | Quartile 1 | 1 | - | 1 | - |
|  | Quartile 2 | 1.29 (0.8-2.1) | - | 1.44 (0.7-2.9) | - |
|  | Quartile 3 | 1.29 (0.8-2.1) | - | 0.91 (0.4-2.2) | - |
|  | Quartile 4 | 1.24 (0.8-2.0) | - | 0.90 (0.4-2.3) | - |

^1^ Presence of an itchy rash at any time in the past 12 months affecting the following places: the folds of the elbows; behind the knees; in front of the ankles; under the buttocks; or around the neck, ears, or eyes

^2^Adjusted by sex and age

^3^ Comparison group: no contact / exposure

^4^Intense truck traffic: Almost the whole day

^5^Disease vectors: fleas, ticks, kissing bugs, mice, bedbugs, flies

^6^ Precarious floor: soil; Precarious walls: cane, palm or trunk; Precarious source of water for cooking: river, lake, pump or water tank car; Precarious sewage system: to the surface or to a septic tank

^7^ Item ownership: Radio, TV, bicycle, motorbike, car, refrigerator, telephone
